# Frameless VarioGuide biopsy for pediatric brainstem lesions in the molecular era: diagnostic performance and workflow characteristics in a comparative single-center series

**DOI:** 10.1007/s00381-026-07484-6

**Published:** 2026-09-30

**Authors:** Sepide Kashefiolasl, Karsten Lachner, Christian J. Braun, Katharina J. Weber, Luciana Porto, Marcus Czabanka, Tobias Finger

**Affiliations:** 1 Department of Neurosurgery, University Hospital Frankfurt, Goethe University, Frankfurt am Main, Germany; 2 Institute of Neuroradiology, University Hospital Frankfurt, Goethe University, Frankfurt am Main, Germany; 3 Department of Pediatrics, University Hospital Frankfurt, Goethe University, Frankfurt am Main, Germany; 4 Institute of Neurology (Edinger Institute), University Hospital Frankfurt, Goethe University, Frankfurt am Main, Germany; 5 German Cancer Consortium (DKTK), Heidelberg, Germany; 6 German Cancer Research Center (DKFZ), Heidelberg, Germany; 7 Frankfurt Cancer Institute (FCI), Goethe University Frankfurt, Frankfurt am Main, Germany; 8 University Cancer Center Frankfurt (UCT), University Hospital Frankfurt, Goethe University, Frankfurt am Main, Germany

**Keywords:** Pediatric brainstem lesions, Brainstem biopsy, VarioGuide, Molecular diagnosis, Pediatric neurosurgery

## Abstract

**Background:**

Molecular characterization has become essential for the diagnosis and treatment of pediatric brainstem lesions. While frame-based stereotactic and robotic-assisted biopsies are well established, comparative data on frameless VarioGuide biopsy for pediatric brainstem lesions remain scarce. This study evaluated the diagnostic performance, safety, and workflow characteristics of frameless VarioGuide biopsy compared with frame-based stereotactic and ROSA-assisted techniques.

**Methods:**

We retrospectively reviewed all pediatric patients (≤ 18 years) who underwent stereotactic biopsy of a brainstem lesion at a single tertiary neurosurgical center between 2016 and 2026. Patients were grouped according to biopsy technique (frameless VarioGuide, frame-based stereotaxy, or ROSA-assisted biopsy). Diagnostic yield, molecular diagnostic yield, complications, operative times, and workflow parameters were analyzed.

**Results:**

Twenty-two pediatric patients met inclusion criteria. Six underwent frameless VarioGuide biopsy, nine frame-based stereotactic biopsy, and seven ROSA-assisted biopsy. Overall diagnostic and molecular diagnostic yields were 95%. Diagnostic and molecular diagnostic yields were 100% with VarioGuide, 89% with frame-based stereotactic biopsy, and 100% with ROSA-assisted biopsy, without statistically significant differences between groups (*p* = 0.62). Median operative times were similar among groups (32, 36, and 25 min, respectively; *p* = 0.28). However, preoperative setup time differed significantly, with the shortest duration observed in the VarioGuide cohort (40.5 min) compared with ROSA-assisted biopsy (64 min) and frame-based stereotaxy (112 min; *p* = 0.002). Despite the absence of intraoperative neuropathological assessment, all VarioGuide procedures yielded diagnostically and molecular-pathologically informative tissue. No procedure-related hemorrhage, infection, permanent neurological deficit, or procedure-related mortality were observed.

**Conclusions:**

In this exploratory single-center cohort, frameless VarioGuide biopsy achieved high diagnostic and molecular diagnostic yields without permanent procedure-related morbidity and was associated with shorter preoperative setup time. These findings support further evaluation of frameless VarioGuide biopsy as a workflow-efficient approach for tissue acquisition in pediatric brainstem lesions.

## Introduction

Brainstem lesions in children comprise a heterogeneous group of pathologies ranging from diffuse midline glioma, H3K27-altered (DMG), and other gliomas to inflammatory and non-neoplastic lesions. Historically, treatment decisions were frequently based on clinical presentation and radiographic findings alone because of concerns regarding the safety of brainstem biopsy. However, advances in stereotactic neurosurgery, perioperative imaging, and molecular neuropathology have shifted clinical practice toward tissue-based diagnosis. In the current World Health Organization (WHO) Classification of Central Nervous System Tumors, molecular alterations play a central role in diagnosis, prognostication, and treatment selection, making tissue acquisition increasingly important in pediatric neuro-oncology [1, 2].

Multiple studies have demonstrated that stereotactic biopsy of pediatric brainstem lesions is safe and associated with high diagnostic yield [3–8]. Contemporary series report diagnostic rates exceeding 90% with low rates of permanent neurological morbidity and procedure-related mortality [3–8]. Furthermore, histopathological diagnosis obtained by stereotactic biopsy has been shown to substantially influence subsequent therapeutic decision-making in pediatric patients [8]. Consequently, stereotactic biopsy has become an integral component of the diagnostic workup of pediatric brainstem lesions, particularly in the context of molecularly guided therapies and clinical trials [4, 6, 9].

Various stereotactic platforms are currently available for brainstem biopsy. Conventional frame-based stereotactic techniques remain widely used and provide excellent targeting accuracy. More recently, robotic-assisted systems such as ROSA have demonstrated similarly favorable safety and diagnostic outcomes [10, 11]. Frameless neuronavigation-guided techniques, including the VarioGuide system, offer an alternative approach that avoids stereotactic frame placement while maintaining navigational accuracy.

While diagnostic yield and procedural safety have been extensively investigated, comparatively little attention has been paid to workflow efficiency and perioperative logistics. In pediatric patients, prolonged anesthesia times, additional imaging requirements, stereotactic frame placement, and complex registration procedures may increase procedural burden. Frameless stereotactic approaches may simplify workflow, reduce preoperative setup time, and facilitate combined procedures such as cerebrospinal fluid diversion during the same surgical session.

Because brainstem lesions represent the anatomically most challenging and highest-risk indication for stereotactic biopsy in pediatric neurosurgery, the present study specifically focused on this distinct subgroup. The aim of the present study was to evaluate the safety, diagnostic performance, and procedural characteristics of frameless VarioGuide biopsy in pediatric brainstem lesions and to compare its performance with frame-based stereotactic and ROSA-assisted biopsy techniques. We hypothesized that frameless VarioGuide biopsy would achieve high diagnostic and molecular diagnostic yield while reducing procedural setup requirements.

## Methods

### Study population

A retrospective review of a prospectively maintained institutional database was performed to identify all pediatric patients (≤ 18 years) who underwent stereotactic biopsy of a brainstem lesion at University Hospital Frankfurt between 2016 and 2026. There was no patient overlap between the present cohort and the previously published institutional series by Quick-Weller et al. [8] Brainstem lesions involving the midbrain, pons, or medulla oblongata were eligible for inclusion, as the present study specifically focused on evaluating stereotactic biopsy techniques in this anatomically challenging location. Patients undergoing open biopsy or with incomplete clinical or radiological documentation precluding retrospective analysis were excluded.

Demographic, radiological, surgical, pathological, and follow-up data were extracted from electronic medical records. Patients were categorized according to the biopsy technique used: frameless VarioGuide stereotactic biopsy, frame-based stereotactic biopsy (STX), or ROSA-assisted robotic biopsy. The choice of stereotactic platform primarily reflected institutional practice and technology availability during the study period rather than predefined lesion-specific selection criteria. Frame-based stereotactic biopsy represented the initial standard approach. Following introduction of the ROSA robotic platform, robotic-assisted biopsies were increasingly performed. After acquisition and institutional implementation of the VarioGuide system, frameless stereotactic biopsies were subsequently introduced. Platform selection was not based on predefined tumor-specific characteristics such as lesion location, size, or the presence of hydrocephalus.

### Preoperative magnetic resonance imaging

All patients underwent preoperative magnetic resonance imaging (MRI) as part of the routine diagnostic workup before stereotactic biopsy. The institutional pediatric brain tumor imaging protocol evolved during the study period and was updated over time; in later years, the protocol was aligned with the 2023 recommendations of the Children’s Oncology Group (COG), Society for Pediatric Radiology (SPR), and American Society of Pediatric Neuroradiology (ASPNR) for standardized pediatric brain tumor imaging [12]. The protocol included high-resolution three-dimensional pre- and post-contrast T1-weighted imaging, axial and sagittal T2-weighted imaging, fluid-attenuated inversion recovery (FLAIR), diffusion-weighted imaging (DWI), susceptibility-weighted imaging (SWI), and multiplanar post-contrast sequences for neuronavigation and trajectory planning. In all but one patient, MRI was performed within 7 days before surgery, and the most recent preoperative examination served as the basis for stereotactic trajectory planning and neuronavigation.

### Surgical technique and workflow assessment

All procedures were performed under general anesthesia by the senior pediatric neurosurgical team. The choice of biopsy technique was based on the stereotactic platform available and institutional practice at the time of surgery.

For frame-based stereotactic biopsies, a Leksell stereotactic frame (Elekta AB, Stockholm, Sweden) was applied after induction of anesthesia. Subsequently, patients underwent stereotactic computed tomography (CT) imaging, which was fused with preoperative magnetic resonance imaging (MRI) for trajectory planning. Following target calculation and trajectory planning, the biopsy procedure was performed using the stereotactic frame system.

For ROSA-assisted biopsies, preoperative planning was performed using the ROSA robotic platform (Zimmer Biomet, Montpellier, France). After patient positioning, registration was achieved using facial surface registration before robotic guidance and tissue acquisition.

**Fig. 1:**
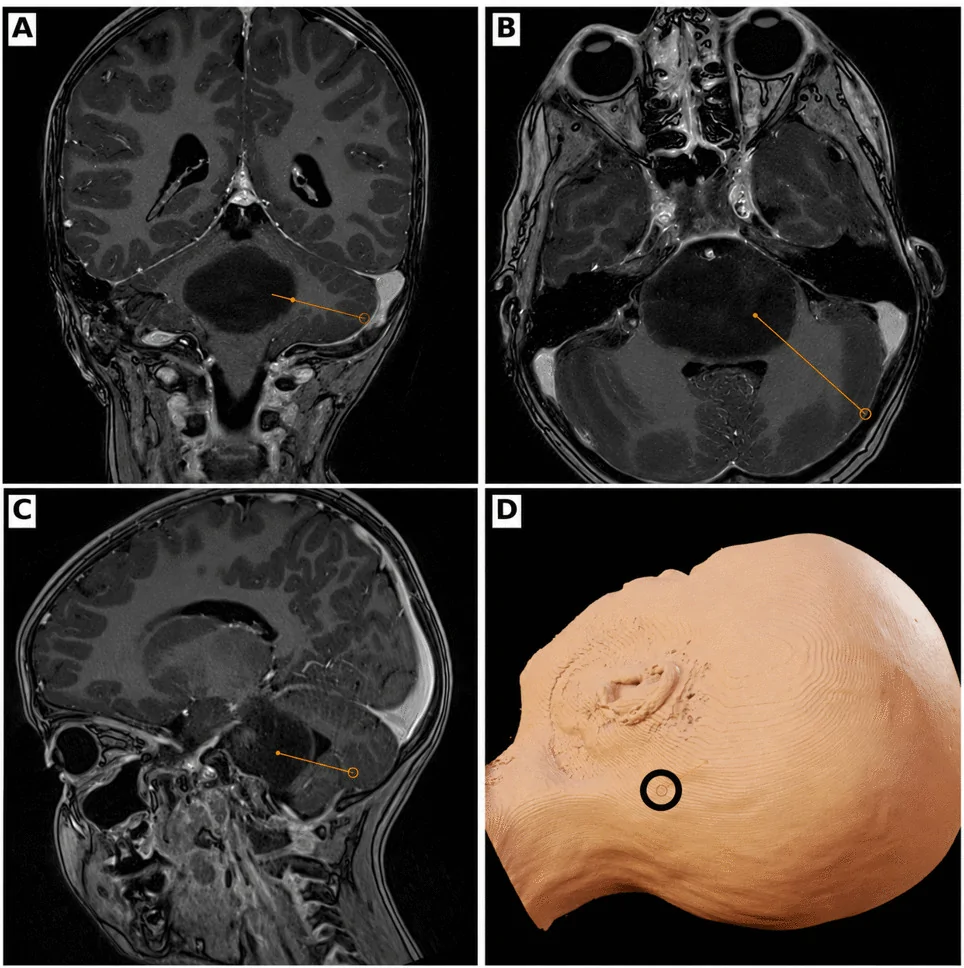
Representative VarioGuide-assisted trajectory planning for biopsy of a pediatric pontine lesion. **A** Coronal, **B** axial, and **C** sagittal contrast-enhanced T1-weighted magnetic resonance images demonstrating a planned transcerebellar trajectory for frameless VarioGuide-assisted biopsy. The trajectory was planned to reach the lesion while avoiding critical neurovascular structures. **D** Three-dimensional surface reconstruction demonstrating the corresponding occipital entry point used for frameless stereotactic guidance

For frameless biopsies, trajectory planning and navigation were performed using the VarioGuide system (Brainlab AG, Munich, Germany). Following image registration and setup of the navigation-guided biopsy arm, the planned trajectory was verified. Tissue sampling was performed using the disposable pre-calibrated biopsy needle (2.1-mm outer diameter; Brainlab AG, Munich, Germany), which integrates directly into the VarioGuide navigation workflow. Aspiration was applied using an attached syringe during tissue acquisition. A representative example of frameless VarioGuide-assisted trajectory planning for a pediatric pontine lesion is shown in Fig. 1.

Quantitative registration or target registration error was not routinely recorded. Immediate postoperative CT or MRI for trajectory verification was not routinely performed in neurologically stable patients; postoperative imaging was obtained when clinically indicated, particularly in the presence of new neurological symptoms.

Preoperative setup time was defined as the interval between induction of general anesthesia and skin incision and included stereotactic frame placement, image acquisition, image fusion, registration procedures, navigation setup, robotic registration, and surgical preparation, as applicable. Operative time was defined as the interval from skin incision to completion of skin closure (skin-to-skin time).

Biopsy specimens were obtained according to the respective institutional sampling workflow. VarioGuide biopsies were performed using targeted core sampling from the planned intralesional target, typically yielding three core specimens. In contrast, frame-based stereotactic and ROSA-assisted biopsies were performed using serial sampling along the planned trajectory, resulting in a larger number of individual small tissue specimens. Thus, the number of recorded specimens reflects differences in sampling strategy and should not be interpreted as a direct measure of total tissue volume.

Intraoperative neuropathological assessment was defined as immediate on-site evaluation of biopsy material by a neuropathologist to confirm lesional tissue acquisition during the procedure. This assessment was routinely performed during frame-based STX and ROSA-assisted biopsies but not during VarioGuide procedures.

### Management of hydrocephalus and simultaneous CSF diversion

The indication for cerebrospinal fluid (CSF) diversion was determined individually based on clinical symptoms, radiological findings, and institutional practice at the time of treatment. Although endoscopic third ventriculostomy (ETV) represents an alternative treatment option for selected patients with obstructive hydrocephalus, no ETV was performed in the present cohort. In patients presenting with symptomatic hydrocephalus requiring urgent treatment, ventriculoperitoneal shunt (VPS) implantation was performed before stereotactic biopsy.

For patients undergoing ROSA-assisted biopsy with a concomitant indication for CSF diversion, VPS implantation was performed during the same surgical session after completion of the biopsy. Owing to the robotic setup, patient repositioning and repeat sterile preparation were required.

For patients undergoing frame-based stereotactic biopsy, VPS implantation was generally performed as a separate operation owing to workflow constraints associated with frame-based stereotactic biopsy.

In patients undergoing frameless VarioGuide biopsy, positioning was planned to facilitate both procedures within a single operative setup. Patients were positioned with ipsilateral shoulder elevation and contralateral head rotation, allowing access to both the transcerebellar biopsy trajectory and the frontal VPS entry site. This strategy enabled stereotactic biopsy and VPS implantation to be performed sequentially during the same surgical session without patient repositioning, repeat sterile preparation, or additional draping.

### Histopathological and molecular analysis

Biopsy specimens were evaluated by experienced neuropathologists according to the World Health Organization (WHO) Classification of Central Nervous System Tumours applicable at the time of diagnosis (4th and 5th editions during the study period). Histopathological diagnoses were established using routine microscopic examination and immunohistochemical analyses.

Molecular testing included immunohistochemical, targeted molecular, and sequencing-based analyses according to the diagnostic standards and tissue availability at the time of surgery. Immunohistochemical assessment of H3K27-altered status formed part of the routine diagnostic workup for pediatric brainstem lesions. Additional molecular investigations were performed when clinically indicated and in accordance with the contemporaneous WHO classification criteria and institutional diagnostic protocols. Final integrated WHO diagnoses were established according to the WHO Classification applicable at the time of diagnosis, incorporating histopathological findings and available molecular data. DNA methylation profiling was performed in selected cases when considered diagnostically relevant and sufficient tissue was available.

Diagnostic biopsy yield was defined as acquisition of sufficient tissue to establish a definitive neuropathological diagnosis according to the contemporaneous WHO classification and diagnostic standards. Molecular diagnostic yield was defined as acquisition of sufficient tissue for molecular characterization. Integrated WHO diagnosis was defined as establishment of a final diagnosis incorporating histopathological and molecular findings according to the applicable WHO classification criteria at the time of diagnosis.

### Outcome measures

The primary outcome measure was diagnostic biopsy yield. Secondary outcome measures included molecular diagnostic yield, integrated WHO diagnosis, operative time, preoperative setup time, hospital length of stay, need for repeat biopsy, and procedure-related complications.

Procedure-related complications included hemorrhage, infection, cerebrospinal fluid leak, wound complications, new neurological deficits, unplanned intensive care unit admission, and procedure-related mortality. Disease-related mortality was recorded separately and was defined as death attributable to progression of the underlying disease during follow-up.

### Ethics approval

The study was approved by the Ethics Commission of the Medical Faculty of Goethe University Frankfurt am Main, Germany (Approval Number 2026-3056). The requirement for informed consent was waived owing to the retrospective nature of the study and the use of anonymized data.

### Statistical analysis

Statistical analyses were performed using IBM SPSS Statistics version 29 (IBM Corp., Armonk, NY, USA). Continuous variables are presented as medians with interquartile ranges (IQRs) and were compared using the Kruskal–Wallis test. Categorical variables are presented as frequencies and percentages and were compared using Fisher’s exact test. A two-sided *p*-value < 0.05 was considered statistically significant.

## Results

### Patient characteristics

**Fig. 2:**
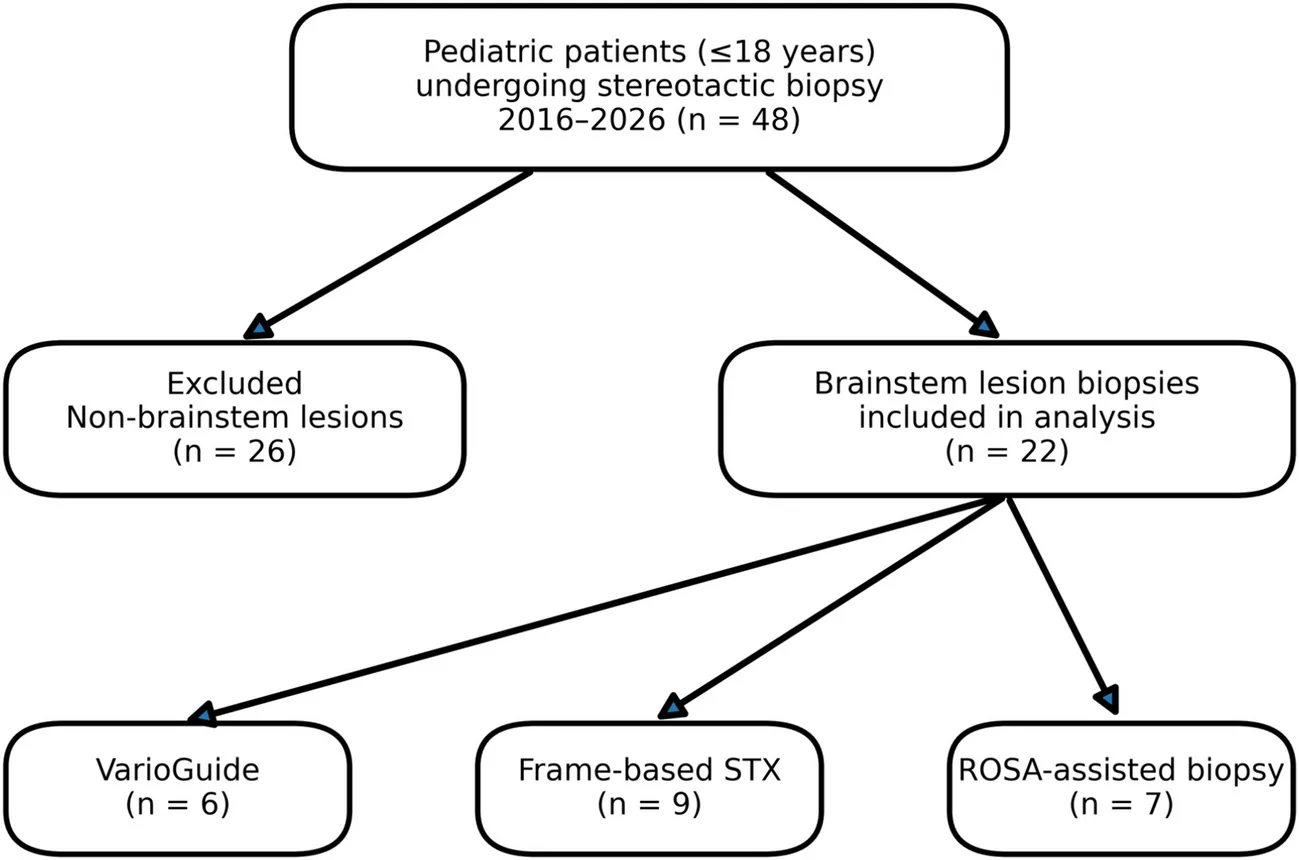
Patient selection flowchart. Selection of the study cohort from all pediatric stereotactic biopsies performed between 2016 and 2026. Twenty-two patients with brainstem lesions met the inclusion criteria and were analyzed according to biopsy technique (VarioGuide, frame-based stereotaxy, and ROSA-assisted biopsy)

A total of 48 pediatric patients underwent stereotactic biopsy during the study period. Twenty-six patients with non-brainstem lesions were excluded. The final study cohort comprised 22 pediatric patients who underwent stereotactic biopsy of a brainstem lesion. The patient selection process is illustrated in Fig. 2. Baseline demographic and clinical characteristics are summarized in Table 1. The median age at diagnosis was 6.5 years, and 12 patients (54.5%) were male. Hydrocephalus requiring CSF diversion was present in 13 patients (59%), and simultaneous VPS placement was performed in 6 patients (27%). No patient underwent ETV. Biopsies were performed using frameless VarioGuide stereotactic biopsy in 6 patients (27%), ROSA-assisted robotic stereotactic biopsy in 7 patients (32%), and frame-based stereotactic biopsy in 9 patients (41%).

**Table 1:** Demographic and clinical characteristics of the pediatric brainstem biopsy cohort

Variable	Ped. cohort (*n* = 22)
Age at diagnosis, median (IQR), years	6.5 (4.25–8.0)
Female sex, *n* (%)	10 (45.5%)
Male sex, *n* (%)	12 (54.5%)
Hydrocephalus requiring CSF diversion, *n* (%)	13 (59%)
Simultaneous VPS placement, *n* (%)	6 (27%)
Frameless Vario Guide biopsy, *n* (%)	6 (27%)
ROSA robotic biopsy, *n* (%)	7 (32%)
Frame-based stereotactic biopsy, *n* (%)	9 (41%)
Open biopsy, *n* (%)	0 (0%)
Operative time (skin-to-skin), median (IQR), min	34 (23.25–38.5)
Length of hospital stay, median (IQR), days	7 (5.25–10.5)

Continuous variables are reported as median (IQR). Categorical variables are reported as *n* (%)

Median operative time was 34 min, and median length of hospital stay was 7 days (Table 1).

### Procedural characteristics

Procedural characteristics according to biopsy technique are summarized in Table 2. Median skin-to-skin operative times were 32, 36, and 25 min for VarioGuide, frame-based stereotactic biopsy, and ROSA-assisted biopsy, respectively (*p* = 0.28; Table 2). In contrast, preoperative setup time differed significantly between techniques, with a median of 40.5 min (IQR 34.25–46.75) for VarioGuide, 64 min (IQR 48.5–95.5) for ROSA-assisted biopsy, and 112 min (IQR 100–130) for frame-based stereotactic biopsy (*p* = 0.002). Intraoperative neuropathological assessment was performed in all frame-based STX and ROSA-assisted procedures, whereas no intraoperative neuropathological assessment was performed in the VarioGuide cohort.

**Table 2:** Procedural characteristics and workflow metrics according to biopsy technique

Variable	VarioGuide (*n* = 6)	Frame-based STX (*n* = 9)	ROSA (*n* = 7)	*p*-value
Year of surgery	2022–2026	2016–2022	2019–2025	-
Age at diagnosis, median (IQR), years	5.5 (IQR 5.0–6.75)	8 (7–14)	5 (4–6)	-
Operative time (skin-to-skin) median (IQR), min	32 (23.75–38)	36 (25–39)	25 (17–34.5)	0.28
Preoperative setup time (anesthesia induction to skin incision), median (IQR), min	40.5 (34.25–46.75)	112 (100–130)	64 (48.5–95.5)	**0.002**
Intraoperative neuropathological assessment, *n* (%)	0 (0%)	9 (100%)	7 (100%)	**-**
Hospital stay, median (IQR), days	5.5 (3.25–8.5)	6 (6–8)	9 (7–13.5)	0.46
Number of biopsy specimens obtained	3 core biopsy specimens	12 serial biopsy specimens (1 mm)	11 serial biopsy specimens (1 mm)	-
Hydrocephalus present, *n* (%)	4 (67%)	5 (56%)	4 (57%)	0.90
Simultaneous VPS placement, *n* (%)	2 (33%)	1 (11%)	3 (43%)	0.34

Continuous variables are presented as median (IQR), and categorical variables as frequencies (percentages). Group comparisons were performed using the Kruskal–Wallis test for continuous variables and Fisher's exact test for categorical variables. A *p*-value < 0.05 was considered statistically significant. Intraoperative neuropathological assessment refers to immediate on-site evaluation of biopsy material by a neuropathologist to confirm lesional tissue acquisition during the procedure. The number of biopsy specimens was not statistically compared because sampling was protocol-driven and differed qualitatively between techniques (targeted core sampling with VarioGuide versus serial sampling with frame-based STX and ROSA); specimen counts therefore do not represent directly equivalent measures of tissue volume

Tissue sampling strategies differed substantially between techniques. VarioGuide biopsies were performed using a median of three core biopsy specimens, whereas frame-based stereotactic and ROSA-assisted procedures yielded a median of 12 and 11 serial biopsy specimens, respectively. Despite the absence of intraoperative neuropathological assessment, all VarioGuide biopsies yielded diagnostically and molecularly informative tissue.

The prevalence of hydrocephalus and the frequency of simultaneous VPS implantation did not differ significantly among groups. In the VarioGuide cohort, biopsy and VPS implantation could be performed within the same positioning and sterile preparation.

### Diagnostic yield and final diagnoses

Diagnostic yield and final diagnoses are summarized in Table 3. Overall diagnostic biopsy yield was 95% (21/22 patients). Diagnostic yield was 100% in the VarioGuide group, 89% in the frame-based stereotactic group, and 100% in the ROSA group, without significant differences between techniques (*p* = 0.62).

**Table 3:** Diagnostic yield and final diagnoses according to biopsy technique

Variable	Ped. cohort (*n* = 22)	Vario guide (*n* = 6)	Frame-based STX (*n* = 9)	ROSA (*n* = 7)	*p*-value
A. Diagnostic yield according to biopsy technique					
Diagnostic biopsy yield	21 (95%)	6 (100%)	8 (89%)	7 (100%)	0.62
Repeat biopsy required	1 (4.5%)	0 (0%)	1 (11%)	0 (0%)	0.74
Molecular diagnostic yield	21 (95%)	6 (100%)	8 (89%)	7 (100%)	0.62
Integrated WHO diagnosis	16 (73%)	5 (83%)	7 (78%)	4 (57%)	0.60
B. Distribution of final diagnoses according to biopsy technique					
Diagnosis	Ped. cohort (*n* = 22)		Vario Guide (*n* = 6)	Frame-based STX (*n* = 9)	ROSA (*n* = 7)
Diffuse midline glioma, H3K27-altered (DMG)	**15 (68%)**		5 (83%)	6 (67%)	4 (57%)
Low-grade glioma (LGG)	**5 (23%)**		0 (0%)	3 (33%)	2 (29%)
High-grade glioma (HGG) (non-DMG)	**1 (4.5%)**		0 (0%)	0 (0%)	1 (14%)
Inflammatory lesion	**1 (4.5%)**		1 (17%)	0 (0%)	0 (0%)

*DMG* diffuse midline glioma, H3K27-altered, *HGG* high-grade glioma, *LGG* low-grade glioma, *STX* frame-based stereotactic biopsy. Diagnostic biopsy yield was defined as acquisition of sufficient tissue to establish a definitive neuropathological diagnosis. Molecular diagnostic yield was defined as acquisition of sufficient tissue for molecular characterization. Low-grade glioma and high-grade glioma refer to the final integrated WHO diagnoses established according to the WHO Classification of Central Nervous System Tumours applicable at the time of diagnosis, incorporating histopathological and available molecular findings. Group comparisons were performed using Fisher’s exact test. A two-sided *p*-value < 0.05 was considered statistically significant

Molecular diagnostic yield was likewise 95% overall and did not differ significantly among groups (*p* = 0.62). Molecular diagnostic adequacy was fully concordant with diagnostic biopsy adequacy: each biopsy that yielded a definitive neuropathological diagnosis also provided sufficient tissue for the molecular characterization performed as part of the clinical diagnostic workup at the time of diagnosis. An integrated WHO diagnosis was established in 16 patients (73%), including 83% of VarioGuide cases, 78% of frame-based stereotactic cases, and 57% of ROSA cases (*p* = 0.60). Only one patient required repeat biopsy.

Diffuse midline glioma, H3K27-altered, CNS WHO grade 4 represented the most frequent diagnosis and was identified in 15 patients (68%). Other diagnoses included low-grade glioma in five patients (23%), non-DMG high-grade glioma in one patient (4.5%), and an inflammatory lesion in one patient (4.5%) (Table 3).

### Safety and clinical outcomes

Procedure-related complications and clinical outcomes are summarized in Table 4. No procedure-related hemorrhage, infection, cerebrospinal fluid leak, wound complication, unplanned intensive care unit admission, or procedure-related mortality occurred. Two patients (9%) experienced a transient neurological deficit, both of whom recovered completely during follow-up. No permanent neurological deficits were observed.

**Table 4:** Procedure-related complications and clinical outcomes

Outcome	Ped. cohort (*n* = 22)	VarioGuide (*n* = 6)	Frame-based STX (*n* = 9)	ROSA (*n* = 7)
Hemorrhage	0 (0%)	0 (0%)	0 (0%)	0 (0%)
Infection	0 (0%)	0 (0%)	0 (0%)	0 (0%)
CSF leak	0 (0%)	0 (0%)	0 (0%)	0 (0%)
Wound complication	0 (0%)	0 (0%)	0 (0%)	0 (0%)
New transient neurological deficit	2 (9%)	0 (0%)	1 (11%)	1 (14%)
Permanent neurological deficit	0 (0%)	0 (0%)	0 (0%)	0 (0%)
Unplanned ICU admission	0 (0%)	0 (0%)	0 (0%)	0 (0%)
Procedure-related mortality	0 (0%)	0 (0%)	0 (0%)	0 (0%)
Deaths during follow-up, *n* (%)	12 (55%)	2 (33%)	4 (44%)	6 (86%)

*ICU* intensive care unit, *CSF* cerebrospinal fluid. Transient neurological deficits consisted of worsening cranial nerve palsy, ataxia, or hemiparesis and resolved completely within 72 h after surgery. Procedure-related mortality was defined as death attributable to the biopsy procedure. During follow-up, 11 deaths were attributable to tumor progression, whereas one patient with a low-grade glioma died from aspiration pneumonia secondary to pre-existing lower cranial nerve dysfunction. No deaths were procedure-related. Mortality data are descriptive only. Because the stereotactic platforms were used during different time periods, follow-up duration was not uniform across groups; crude mortality proportions should therefore not be interpreted as comparative outcome measures between biopsy techniques

During follow-up, 12 patients (55%) died. Eleven deaths were attributable to progression of the underlying disease, whereas one patient with a low-grade glioma died from aspiration pneumonia related to pre-existing lower cranial nerve dysfunction. No death was related to the biopsy procedure. Because the stereotactic platforms were used during different periods of the study with inherently unequal potential follow-up durations, mortality proportions are presented descriptively and should not be interpreted as comparative outcome measures between biopsy techniques (Table 4).

## Discussion

In this exploratory single-center series, frameless VarioGuide biopsy achieved high diagnostic and molecular diagnostic yields and was associated with significantly shorter preoperative setup time than the other stereotactic techniques evaluated. All six VarioGuide biopsies yielded diagnostically and molecularly informative tissue, with no permanent neurological deficits or procedure-related mortality. These findings indicate favorable diagnostic and workflow characteristics of frameless VarioGuide biopsy in this cohort, while the limited sample size precludes conclusions regarding formal equivalence or superiority between stereotactic platforms.

### Brainstem biopsy in the molecular era

The role of stereotactic biopsy in pediatric brainstem lesions has evolved considerably over the past decade. Historically, many brainstem tumors, particularly diffuse intrinsic pontine gliomas, were diagnosed solely based on clinical presentation and radiographic findings because of concerns regarding biopsy-related morbidity. However, advances in molecular neuropathology and the introduction of integrated WHO classifications have substantially increased the value of tissue acquisition for diagnostic confirmation, molecular characterization, prognostication, and enrollment in molecularly guided clinical trials [1, 6, 9].

Previous studies have consistently demonstrated that stereotactic biopsy of pediatric brainstem lesions is associated with high diagnostic yield and low morbidity [3–8, 13]. Recent data from Früh et al. further confirmed the feasibility and safety of biopsy in pediatric caudal brainstem tumors, with sufficient tissue acquisition for integrated neuropathological diagnosis in the vast majority of patients [13]. In particular, Quick-Weller et al. reported low biopsy-related morbidity in a pediatric brainstem cohort and highlighted the substantial impact of histopathological diagnosis on subsequent treatment decisions [8]. While histopathological confirmation alone previously represented a major clinical benefit, the contemporary molecular era has further expanded the importance of tissue acquisition. Today, biopsy specimens are frequently required not only for histological diagnosis but also for molecular characterization and integrated WHO classification.

Consistent with previous reports, the overall diagnostic yield in the present cohort was high, with diagnostic tissue obtained in 95% of patients and molecular characterization possible in 95% of cases. Importantly, diagnostic and molecular diagnostic yields were high across all three stereotactic approaches, including 100% diagnostic and molecular diagnostic yield in the VarioGuide cohort, despite the different sampling strategy used with this technique.

In the present cohort, a limited number of targeted core specimens was sufficient for the intended clinical histopathological and molecular diagnostic workup in all VarioGuide cases. However, this observation should not be interpreted as establishing three cores as a generally sufficient sampling strategy. Contemporary clinical trials and translational studies may require additional tissue for extended genomic profiling, cell culture, functional drug testing, or other investigational analyses. Tissue sampling should therefore be adapted to the intended diagnostic and research requirements while balancing tissue requirements against procedural safety in this anatomically eloquent location.

### Workflow efficiency and procedural burden

The most notable finding of the present study was the significant reduction in preoperative setup time associated with the VarioGuide technique. Median setup time was 40.5 min for VarioGuide compared with 64 min for ROSA-assisted biopsy and 112 min for frame-based stereotactic biopsy. These differences likely reflect a combination of platform-specific workflow requirements and institutional factors. In particular, frame-based stereotaxy requires additional steps including frame placement, stereotactic CT acquisition, and image fusion, whereas differences between the two frameless platforms may also have been influenced by institutional familiarity, operator experience, and workflow optimization.

Notably, VarioGuide biopsies were performed without intraoperative neuropathological assessment, whereas immediate on-site tissue evaluation was available during frame-based STX and ROSA-assisted procedures. Despite this difference, diagnostic and molecular diagnostic yield remained 100% in the VarioGuide cohort. Although intratumoral molecular heterogeneity has been described in diffuse midline gliomas, H3K27-altered, potentially affecting the detection of secondary molecular alterations, all VarioGuide biopsies in the present cohort yielded sufficient tissue for the intended histopathological and molecular analyses [14]. These findings suggest that careful trajectory planning and targeted core sampling can provide diagnostically representative tissue despite obtaining only a limited number of biopsy specimens and without immediate neuropathological confirmation.

Although stereotactic accuracy remains the primary objective of any biopsy procedure, workflow efficiency represents an increasingly relevant consideration in pediatric neurosurgery. Children undergoing brainstem biopsy require general anesthesia, and minimizing non-therapeutic procedural steps may reduce overall procedural burden. Furthermore, streamlined workflows may improve operating room efficiency and facilitate combined procedures when clinically indicated.

Frame-based stereotactic procedures inherently require several additional preparation steps, including frame placement, stereotactic imaging, image fusion, and trajectory calculation. Previous technical analyses have highlighted the importance of frame positioning and trajectory optimization for successful stereotactic targeting, illustrating the complexity of conventional stereotactic workflows [15]. In contrast, frameless neuronavigation-guided approaches eliminate several of these preparatory steps while maintaining satisfactory targeting accuracy.

An additional practical workflow consideration observed in the present series was the possibility of performing VPS placement during the same surgical session. In patients in whom VPS placement was clinically indicated, the VarioGuide setup used at our institution allowed biopsy and shunt implantation without patient repositioning or repeat sterile preparation. Although this may facilitate combined procedures in selected patients, this observation applies specifically to VPS placement and should not be interpreted as a general advantage of the frameless technique for hydrocephalus management, as ETV may represent an alternative in appropriately selected patients.

To our knowledge, few studies have specifically investigated workflow-related parameters when comparing stereotactic biopsy platforms for pediatric brainstem lesions. Most published series have focused primarily on diagnostic yield, targeting accuracy, and complication rates rather than workflow-related metrics [3–8, 10–12, 15–17]. Our findings suggest that procedural efficiency may represent an additional factor when selecting an appropriate stereotactic platform.

### Safety of frameless stereotactic biopsy

Safety remains the most important consideration when performing biopsy of eloquent brainstem structures. In the present cohort, no procedure-related hemorrhage, infection, cerebrospinal fluid leak, wound complication, unplanned intensive care unit admission, or procedure-related mortality occurred. Two patients experienced transient neurological deficits that resolved completely during follow-up, and no permanent neurological deficits were observed.

These findings are consistent with contemporary reports demonstrating low morbidity and mortality rates following stereotactic biopsy of pediatric brainstem lesions [3–8]. Similarly, Früh et al. reported a favorable safety profile for biopsies of pediatric caudal brainstem tumors, with no procedure-related mortality and a high diagnostic success rate, further supporting the safety of contemporary stereotactic biopsy techniques in eloquent brainstem locations [13]. Previous studies have reported permanent neurological deficit rates ranging from 0% to approximately 5%, whereas procedure-related mortality has become exceedingly rare in modern stereotactic series [3–8]. Notably, previous experience from our institution similarly demonstrated low morbidity and a substantial impact of biopsy results on treatment decisions in children with brainstem lesions [8]. The present study extends these observations by describing the diagnostic, safety, and workflow characteristics of frameless VarioGuide biopsy in this pediatric cohort. Future integration of advanced MRI techniques and radiomics-based molecular prediction models may further optimize biopsy target selection, trajectory planning, and individualized management of pediatric brainstem lesions [18].

### Limitations

The lower rate of final integrated WHO diagnosis compared with molecular diagnostic yield reflects the retrospective study period spanning 2016 –2026, during which molecular testing algorithms and WHO classification criteria evolved substantially. In some earlier cases, tissue was sufficient for clinically relevant molecular characterization but not for complete integrated classification according to current WHO criteria.

Several limitations should be considered when interpreting the present findings. First, this study represents a retrospective single-center analysis with a limited sample size. Consequently, the study was not powered to detect small differences in diagnostic yield or complication rates between biopsy techniques. Second, the choice of stereotactic platform was influenced by institutional practice and technological availability over time, introducing potential selection bias. Frame-based stereotactic biopsy represented the historical standard approach, whereas ROSA-assisted and subsequently VarioGuide-guided procedures were introduced later during the study period. Consequently, differences in workflow may partially reflect institutional familiarity, operator experience, platform-specific learning curves, and procedural optimization in addition to inherent differences between the stereotactic systems. The observed setup time differences should therefore be interpreted as an association within our institutional cohort and may not be directly generalizable to centers with different levels of experience with the respective platforms.

Third, the study period spanned a decade from 2016 to 2026, during which molecular diagnostic algorithms, availability of DNA methylation profiling, and WHO classification criteria evolved considerably. Consequently, molecular analyses were not entirely uniform across all patients, and some earlier cases could not be retrospectively assigned a complete integrated WHO diagnosis despite successful molecular characterization.

Furthermore, advanced MRI techniques such as diffusion tensor imaging (DTI), perfusion MRI, magnetic resonance spectroscopy (MRS), or amide proton transfer (APT)-weighted imaging were not uniformly available throughout the study period and were therefore not routinely incorporated into stereotactic trajectory planning. Future integration of advanced imaging techniques may further optimize target selection and workflow in pediatric brainstem biopsy.^15^

Quantitative registration and targeting accuracy data were not systematically available for the VarioGuide procedures, and routine immediate postoperative imaging for trajectory verification was not performed; therefore, formal retrospective assessment of targeting accuracy was not possible.

Finally, the relatively small number of patients precludes definitive conclusions regarding superiority of one stereotactic platform over another. Because of the small sample size, particularly within the VarioGuide subgroup, the present study should be interpreted as an exploratory feasibility and workflow analysis rather than evidence of formal non-inferiority between stereotactic platforms.

## Conclusions

In this exploratory single-center cohort, frameless VarioGuide biopsy achieved high diagnostic and molecular diagnostic yields without permanent procedure-related morbidity and was associated with shorter preoperative setup time than the other stereotactic techniques evaluated. Given the limited sample size and retrospective design, these findings should be interpreted as preliminary and do not establish equivalence or superiority between stereotactic platforms. Further studies in larger cohorts are warranted to evaluate the diagnostic and workflow characteristics of frameless stereotactic biopsy in pediatric brainstem lesions.

## Data Availability

The datasets analyzed during the current study are available from the corresponding author on reasonable request, subject to institutional and ethical regulations.
